# Laparoscopic repair of perineal hernia and unilateral inguinal hernia after rectal cancer surgery: A case report

**DOI:** 10.1097/MD.0000000000037223

**Published:** 2024-02-23

**Authors:** Chuan-Ying Li, Hao-Jun Zhao, Yuan Zhou, Jia-You Xu

**Affiliations:** a Weifang Medical University, Weifang, Shandong 261053, China; b Department of General Surgery, Weifang People’s Hospital, Weifang, Shandong 261041, China

**Keywords:** abdominoperineal resection, laparoscopic repair, Mesh repair, perineal hernia, surgical approach

## Abstract

**Introduction:**

Perineal hernia (PH) is a rare complication that can occur after abdominoperineal resection for rectal cancer. Laparoscopic repair of PHs has gained increasing popularity compared to open approaches due to advantages such as superior visualization, decreased invasiveness, and faster recovery. This case report highlights the successful use of laparoscopic tension-free mesh repair for concurrent perineal and inguinal hernias after rectal cancer surgery.

**Case Description:**

A 51-year-old man underwent laparoscopic-assisted abdominoperineal resection for rectal cancer. About 2 months postoperatively, he developed reducible masses in the perineal and left groin regions, associated with urinary symptoms and sensation of prolapse. Physical exam revealed protruding masses that enlarged with Valsalva. Pelvic CT confirmed PH and left inguinal hernia.

**Interventions:**

Laparoscopic tension-free repair of the PH and inguinal hernia was performed on this patient. The repair was completed by the steps of adhesion separation, mesh placement, and fixation.

**Outcomes:**

The 98-minute surgery was successful without complications. The patient recovered well, ambulating on postoperative day 2 and getting discharged on day 6.

**Conclusion:**

This case demonstrates that laparoscopic tension-free repair with mesh is an effective approach for treating PH and concurrent inguinal hernia following rectal cancer surgery, resulting in successful outcomes and low recurrence rates. The laparoscopic technique provides benefits of minimal invasiveness and rapid recovery.

## 1. Introduction

Perineal hernia (PH) is a rare complication after abdominoperineal resection for rectal cancer, with an incidence of about 0.34%.^[1]^ The main treatment for PH typically involves surgical intervention, frequently using mesh repair via an abdominal or perineal approach. Here we report a 51-year-old male patient who developed perineal hernia and left inguinal hernia 2 months after laparoscopic-assisted abdominoperineal resection for rectal cancer. We performed laparoscopic tension-free repair of the perineal hernia and inguinal hernia for him, as described below.

## 2. Case report

**Figure 1.**
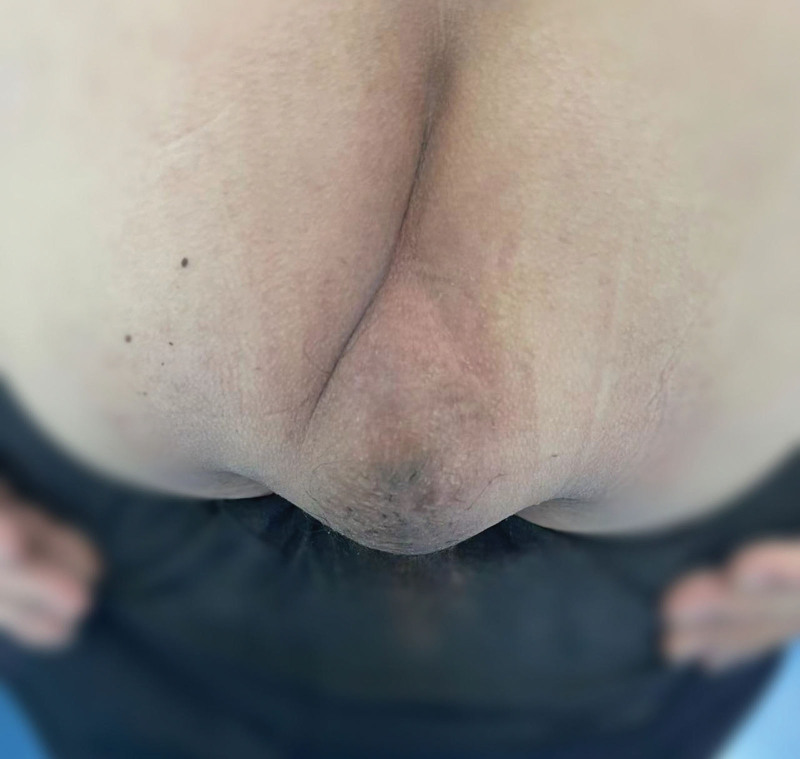
Reversible mass in the perineal area.

**Figure 2.**
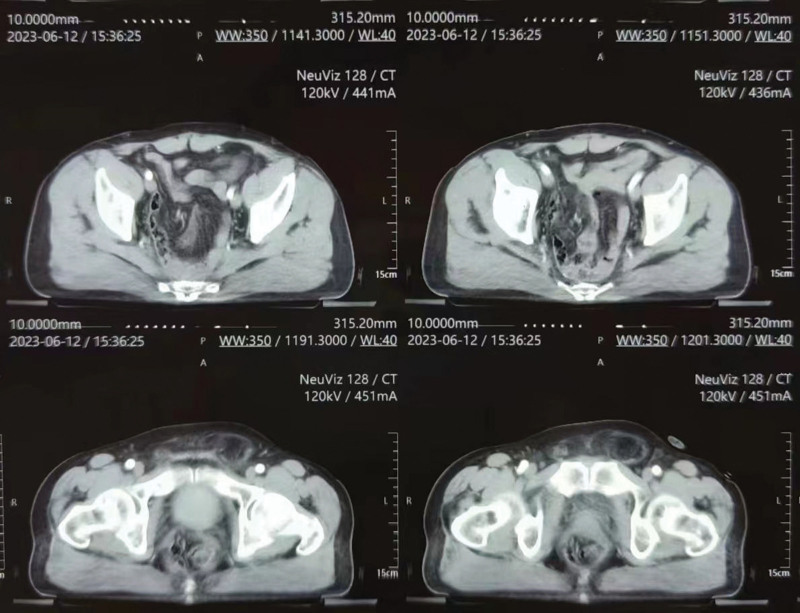
Perineal hernia (PH) was diagnosed on pelvic CT.

**Figure 3.**
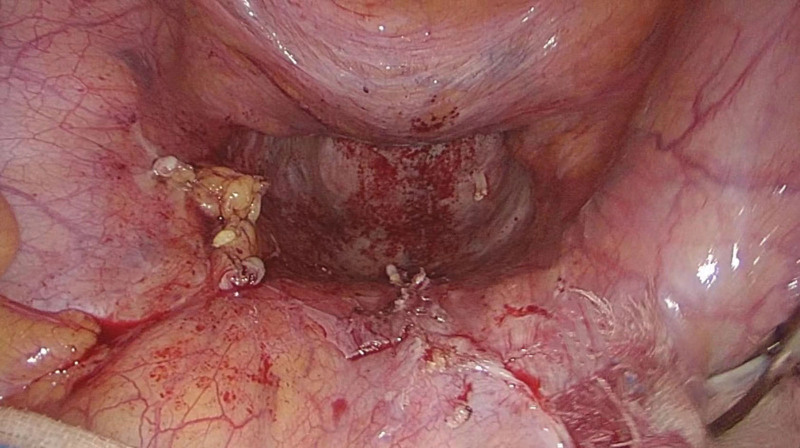
The hernia sac.

**Figure 4.**
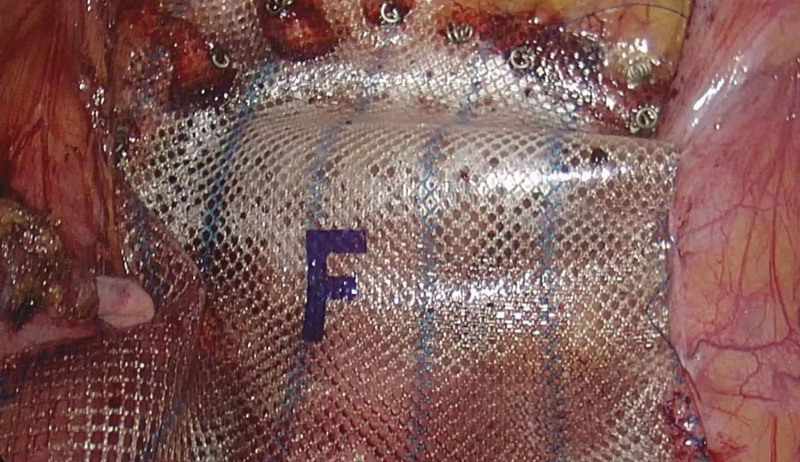
Nonabsorbable tacks fixed the anti-adhesive mesh.

On December 15, 2022, a laparoscopic-assisted abdominoperineal resection was performed on a 51-year-old male patient with rectal cancer. In February 2023, 2 months after the surgery, the patient reported the presence of reducible masses in the perineal (Fig. 1) and left groin regions, as well as difficulty urinating and a sensation of prolapse. A pelvic CT confirmed the diagnosis of PH in combination with left inguinal hernia (Fig. 2). On June 15, 2023, the patient underwent surgical repair of the PH. Notably, adhesion of the small intestine to the abdominal wall was observed during the surgical procedure. The hernia sac, measuring 4 cm in diameter, protruded outward below the pelvic floor muscle defect (Fig. 3), with the colon as its contents. In the surgery, intra-abdominal adhesions were separated using an ultrasonic scalpel. A peritoneal incision was made 2 cm above the inferior epigastric vessels, extending to the pubic symphysis above, to create a cavity for the extraperitoneal space. The pubic symphysis and bilateral pubic inguinal ligaments were fully exposed. Subsequently, the bladder was separated posteriorly, while preserving the integrity of the bilateral ureters and iliac vessels. The extraperitoneal space surrounding the left oblique hernia sac was detached, and a nonabsorbable anti-adhesive mesh measuring 10 × 15 cm was placed into the abdominal cavity (Fig. 4). The mesh extended anteriorly to the pubic symphysis and posteriorly covered the sacrum and pelvis. The anterior part of the mesh was secured to the pubic symphysis using a nonabsorbable tacking device, while the posterior part was sutured to the parietal peritoneum using 3-0 Prolene. Additionally, for the left inguinal hernia, the nonabsorbable anti-adhesive mesh was fixed with nonabsorbable tacks and the gap between the mesh and the upper peritoneum was continuously sutured with barbed lines. The duration of the operation was 98 minutes. The patient was able to ambulate on the second day after the surgery and was discharged on the 6th day.

## 3. Discussion

PH, a rare disease belonging to pelvic floor hernias, can be further categorized into primary and secondary PHs. Primary perineal hernias are typically observed in middle-aged women due to factors such as a wider female pelvis, reduced pelvic floor tension after childbirth, obesity, abdominal effusion, and recurrent pelvic floor infections.^[2]^ On the other hand, secondary perineal hernias primarily arise following pelvic surgery, particularly rectal surgery. Notably, the incidence of perineal hernia has been on the rise in recent years, largely attributed to the increased use of laparoscopic-assisted abdominoperineal resection for rectal cancer.

The pathogenesis of PH includes various factors. Firstly, congenital anatomical defects play a role in hernia formation. This involves the formation of a long and deep Douglas pouch during embryonic development, which can lead to hernia formation. When there is an increase in intra-abdominal pressure, the Douglas pouch deepens, causing further downward protrusion of the fascia and promoting hernia formation. Secondly, excessive laxity of the tissues supporting the rectum contributes to PH. This is especially true in cases where there is poor development of the pelvic floor muscles, as it prevents the rectum from maintaining its normal position. Consequently, when intra-abdominal pressure rises, the viscera may protrude from the pelvic floor. Another factor contributing to PH is a history of pelvic surgery, which can lead to decreased pelvic floor tissue tension.^[3]^ Lastly, laparoscopic techniques can also play a role in hernia development. Specifically, they can reduce intraperitoneal adhesions and increase the possibility of intestinal loops slipping into the pelvis and protruding into the perineum.

PHs can be classified into anterior and posterior PHs based on the relationship with the perineal transverse muscle. Anterior PHs involve the anterior protrusion of the perineal transverse muscle, extending towards 1 labium majus. On the other hand, posterior PHs occur when the hernia contents descend to the inferior edge of the gluteus maximus, creating a mass between the bladder and rectum. This particular case represents a typical example of a posterior PH. It is worth noting that the most common hernia contents reported in the literature are the small intestine and greater omentum, with some rare instances involving the bladder, uterus, and colon.^[4]^ Generally, PHs are asymptomatic and can be observed as protruding masses in the perineal area during physical examination. These masses become more prominent when Valsalva maneuvers are performed and can sometimes be reduced. However, PHs can cause complications such as difficult urination and defecation, as well as abdominal and perineal prolapse, along with associated pain. In some cases, these hernias can also lead to rectal compression or even intestinal obstruction, necessitating surgical intervention.

The surgical repair with mesh is the recommended treatment for PH. However, there is no consensus on the optimal surgical approach due to the relative rarity of PHs. The current surgical approaches can be classified based on the surgical route and location, which include the perineal approach, abdominal approach, combined surgery, and laparoscopic surgery. The most commonly used method is the perineal approach due to its simplicity and avoidance of entering the abdominal cavity. Nevertheless, the perineal approach has limitations in terms of exposure and cannot fully eliminate the risk of tumor recurrence. Additionally, securing the mesh, especially anteriorly, poses a technical challenge in the perineal approach. Consequently, the limited surgical area often leads to a high hernia recurrence rate due to inadequate mesh deployment. In contrast, the abdominal approach provides better surgical exposure, allowing adequate mesh deployment and ensuring effective monitoring for tumor recurrence. Moreover, other surgeries can be simultaneously performed during PH repair using the abdominal approach. However, the abdominal route is more invasive and requires a longer postoperative recovery compared to the perineal approach. Currently, laparoscopic surgery can also be performed through the abdominal route, combining the advantages of the abdominal approach with the benefits of minimally invasive access. A study demonstrated that abdominal and laparoscopic surgery resulted in lower PH recurrence rates after repair (8.8% and 11.8%, respectively), compared to the perineal approach with a recurrence rate of 35.6%.^[5]^

In clinical practice, tension-free repair with mesh is a reliable method to reduce recurrence rates. The most commonly used mesh materials are nonabsorbable materials, mainly polypropylene. When selecting a mesh for PH repair after rectal cancer surgery, there are synthetic and biological meshes to choose from. Synthetic meshes have advantages such as high mechanical strength, no immune rejection, and low risk of infection. On the other hand, biological meshes have good biocompatibility and can promote tissue regeneration and vascularization. However, they have weaker mechanical strength and the absorption rate is difficult to control, which leads to a weaker ability to prevent recurrence. Reports show that biological meshes have higher recurrence rates than synthetic meshes in PH repair.^[6]^ Therefore, due to the lack of experience with biological mesh repairs, we still recommend using synthetic mesh.

## 4. Conclusion

The choice of specific surgical methods depends on various factors. These factors include whether the hernia contents can be completely reduced, as well as the need to simultaneously address other diseases and concomitant hernias such as inguinal and stoma hernias. Laparoscopic tension-free hernia repair has several advantages compared to perineal and abdominal surgeries. These advantages include minimal invasiveness, clearer view, simpler manipulation, and fewer complications. Additionally, laparoscopic repair can treat concomitant pelvic diseases simultaneously. The patients who undergo laparoscopic tension-free hernia repair can get out of bed on postoperative day 2, and the recurrence rate is low. As a result, laparoscopic tension-free hernia repair is expected to become the preferred treatment for PH.

## Author contributions

Writing—original draft: Chuan-Ying Li.

Writing—review and editing: Chuan-Ying Li, Hao-Jun Zhao, Yuan Zhou, Jia-You Xu.
